# Electroacupuncture vs topical diclofenac sodium gel for patients with hand osteoarthritis: study protocol for a randomized controlled trial

**DOI:** 10.1186/s13018-022-03125-1

**Published:** 2022-04-12

**Authors:** Weiming Wang, Shudan Yu, Zilin Long, Yan Liu, Yan Yan, Tianheng Sun, Zhishun Liu

**Affiliations:** 1 Department of Acupuncture and Moxibustion, China Academy of Chinese Medical Sciences Guang’anmen Hospital, No. 5 Beixiange, Xicheng District, Beijing, 100053 China; 2 Beijing University of Chinese Medicine, Beijing, 100029 China; 3 Key Laboratory of Chinese Internal Medicine of Ministry of Education, Dongzhimen Hospital, Beijing University of Chinese Medicine, Beijing, 100700 China

**Keywords:** Electroacupuncture, Diclofenac sodium gel, Hand osteoarthritis, Clinical trial

## Abstract

**Background:**

Hand osteoarthritis (OA) is a prevalent joint disorder and a great burden to both patients and society. While electroacupuncture (EA) and topical diclofenac sodium gel (DSG) are both currently used to treat OA, no head-to-head study of EA and topical DSG for hand OA exists. Thus, it remains unknown whether one intervention offers improved outcomes over the other. This study aims to compare the effects of EA and topical DSG in patients with hand OA.

**Methods:**

A total of 108 participants with hand OA according to the American College of Rheumatology criteria will be recruited and randomly assigned to the EA group or topical DSG group with a 1:1 allocation ratio. Participants in the EA group will receive EA treatment thrice weekly for 4 weeks, followed by a 12-week follow-up. In the topical DSG group, topical DSG at a dose of 2 g over the affected joints per hand will be applied four times per day for 4 weeks. The outcomes will be measured at weeks 4, 8, and 16. The primary outcome will be the change in average overall finger joint pain intensity in the dominant hand from baseline to week 4. All outcome variables will be analyzed on an intention-to-treat principle. All statistical tests will be two-sided.

**Discussion:**

This study will help determine which of the two treatment protocols, EA or topical DSG, is more effective for the clinical treatment of hand OA.

*Trial registration* ClinicalTrials.gov identifier: NCT04402047. Registered 16 May 2020, https://clinicaltrials.gov/ct2/show/NCT04402047

## Introduction

Hand osteoarthritis (OA) is a prevalent joint disorder characterized by pain, stiffness, and bony enlargements/swellings of multiple joints, in particular the distal interphalangeal (DIP), proximal interphalangeal (PIP), and first carpometacarpal (CMC) joints [1, 2]. Symptomatic hand OA is estimated to affect 15.9% of women and 8.2% of men in the general population [3] with a variable disease course [4], occurring more frequently in the elderly [3, 5]. The exact pathogenesis of hand OA is unknown but likely to be multifactorial [6]. Clinically, hand OA can be categorized into the following subsets: erosive, thumb-based, interphalangeal joint (with or without nodes) OA and generalized hand OA [7]. In addition to pain and stiffness, patients with hand OA often suffer from reduced grip and pinch strength, decreased range of motion in the involved and noninvolved joints [8], and difficulty performing dexterous tasks [9], resulting in disability in activities of daily living and considerable frustration [10].

At present, few therapeutic options with proven effectiveness are available for hand OA [11]. The effects of non-pharmacologic approaches are modest, and non-steroidal anti-inflammatory drugs (NSAIDs) are commonly used in the pharmacological treatment of patients with hand OA. Because topical NSAIDs have equivalent efficacy and a lower frequency of adverse events (AEs) compared with oral NSAIDs [12, 13], topical NSAIDs have been recommended as the preferred treatment option [14] Diclofenac sodium gel (DSG) is one of the commonly used topical NSAIDs, and studies have shown that it can provide local pain relief for patients with hand OA with reduced systemic exposure [15, 16], potentially reducing the risk of AE. However, the modest efficacy of NSAIDs is challenging for clinicians, and alternative effective therapies are still needed.

Acupuncture is an ancient Chinese therapy that provides beneficial effects. It is effective in a host of pain-related conditions, ranging from low back pain, neck pain, shoulder pain, and migraine to pain from knee OA [17]. The exact mechanisms of the analgesic effects of acupuncture are complex and have been thought to involve endogenous opioid release [18]. At present, there is a considerable amount of research demonstrating the positive effects of acupuncture on pain and function in knee OA [19]. Although hand OA is among the most prevalent OA phenotypes [20], the research on the effects of acupuncture in people with hand OA is very limited.

We have designed this pragmatic randomized trial to investigate the clinical effectiveness and safety of 4-week electroacupuncture (EA) therapy compared to topical DSG for the treatment of hand OA. Our primary hypothesis is that EA will provide greater relief from pain in patients with hand OA compared with topical DSG.

## Methods and design

### Study design

**Fig. 1:**
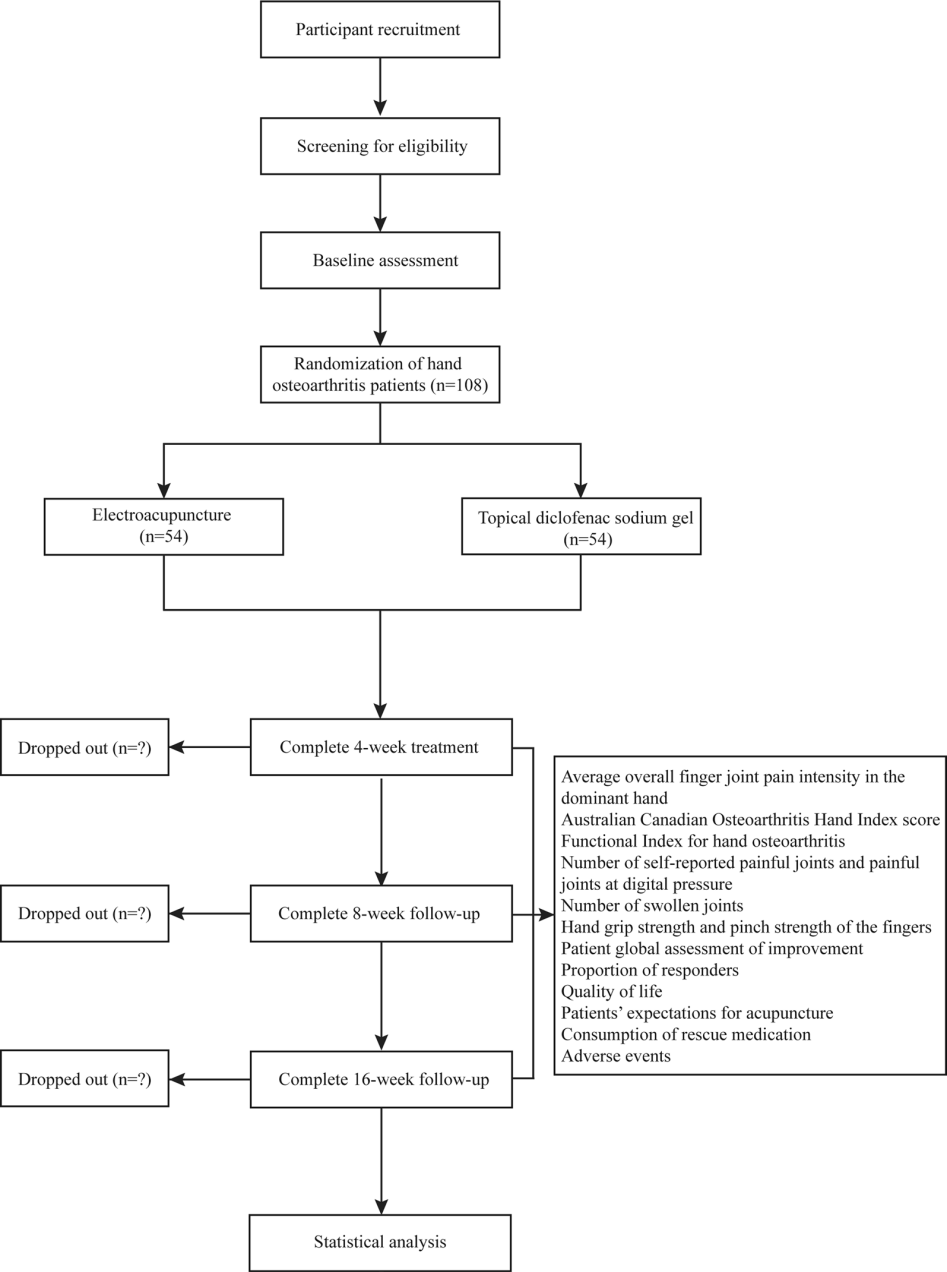
Study flow diagram

**Fig. 2:**
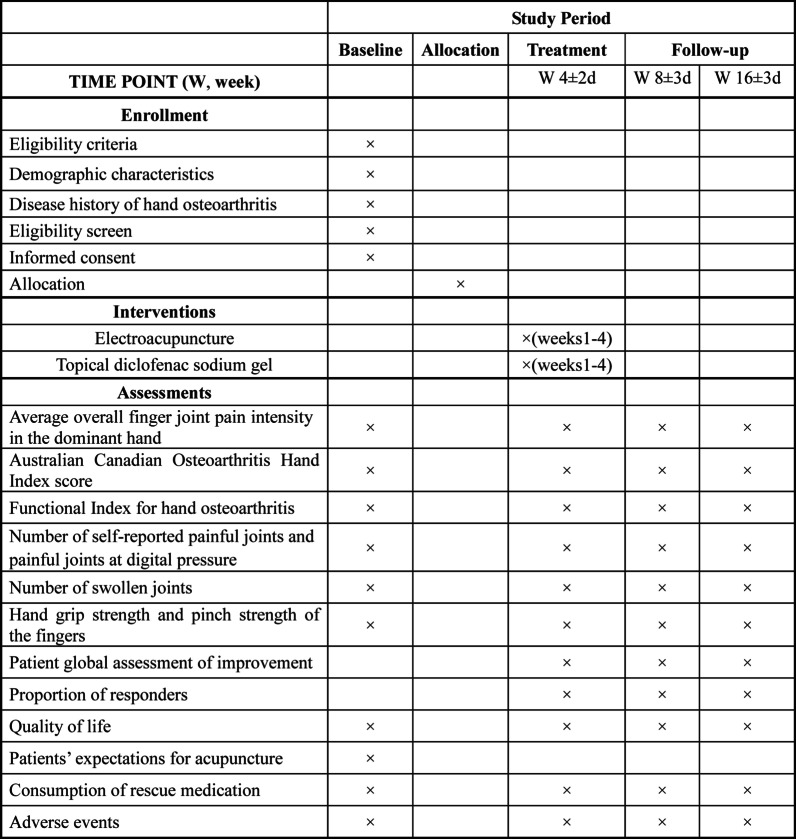
Study schedule

This will be a parallel-design, assessor-blinded, randomized trial with a 1:1 allocation rate. The study is designed to adhere to the standard protocol items including the Recommendations for Interventional Trials [21] and the Standards for Reporting Interventions in Clinical Trials of Acupuncture [22] guidelines. The study flow diagram and treatment schedule are presented in Figs. 1 and 2.

### Study setting and recruitment

This trial will be conducted at Guang’anmen Hospital, China Academy of Chinese Medical Sciences, and Yantai Hospital of Traditional Chinese Medicine from August 2020 to August 2022. A total of 108 participants will be publicly recruited through the use of posters and hospital websites. The duration of the trial for each participant will be 17 weeks: 1-week baseline, 4-week treatment, and 12-week follow-up.

### Randomization and blinding

Participants will be randomized to the EA group or topical DSG group at a ratio of 1:1 using a fixed block randomization (block size *n* = 4). The randomization schedule will be generated with PROC PLAN of SAS 9.4 (SAS Institute Inc., Cary, NC, USA) by the study biostatistician. To help conceal the details of the group allocation, consecutively numbered and sealed opaque envelopes prepared by an independent staff member will be used. After the participant has completed all the baseline measurements, the envelopes will be opened in sequence by a research coordinator who will be not involved in treatment and outcome assessments.

The outcome assessor and data manager will be blinded to the group assignments and not involved in providing the interventions. Allocation will be revealed to the researchers (including acupuncturists) delivering the intervention, but participants will be asked to not disclose details about their group allocation with the outcome assessors.

### Participants

Participants will be considered eligible by a rheumatologist or hand surgeon if they fulfill the American College of Rheumatology (ACR) [23] clinical classification criteria for the diagnosis of hand OA in dominant (target) hand. The criteria are as follows: hand pain, aching or stiffness, and 3 or 4 of the following features: hard tissue enlargement of at least two of 10 selected joints (the 2nd and 3rd DIP and PIP joint and first CMC joint of both hands), hard tissue enlargement of at least two DIP joints, swelling of fewer than three metacarpophalangeal joints, and deformity of at least one of the 10 aforementioned selected joints. For inclusion, they must meet all of the following inclusion criteria and not fulfill any of the exclusion criteria.

### Inclusion criteria

1. Age ≥ 18 years and ≤ 80 years;
2. History of hand OA for at least 3 months before enrollment;
3. Reported an average overall finger joint pain intensity in the dominant hand over the past 48 h of at least 40 mm according to a 100-mm visual analog scale (VAS, 0 = no pain, 100 = maximal pain). Patients applying NSAIDs at the screening must have an increase in pain in the dominant hand of ≥ 20 mm during washout;
4. Posterior–anterior radiographs show Kellgren–Lawrence grade 1, 2, or 3 changes [24] in symptomatic joints;
5. Tests for rheumatoid factor and anticyclonic citrullinated peptide are negative to exclude inflammatory arthritis;
6. Ability to comply with the study protocol, understand the medical information form, and provide written informed consent.

### Exclusion criteria

1. History or current evidence of secondary OA (due to causes other than a solely degenerative joint disease) or symptomatic OA at additional locations besides the hand(s) requiring treatment, or any painful syndrome of the upper limb, which may interfere with evaluation of hand pain;
2. History of inflammatory arthritis (e.g., rheumatoid arthritis [RA], psoriatic arthritis), hemochromatosis, metabolic, or neuropathic arthropathies;
3. History of trauma, dislocation, or operation to the hand or arm in the previous 3 months;
4. Hand pain and stiffness due to tissue scarring or tendinitis;
5. Skin damage or serious skin disorders in the hands;
6. Intake of antidepressants, anticonvulsants, vascular drugs, or narcotics during the 10 days prior to beginning the study;
7. Oral, intramuscular, intra-articular, or intravenous corticosteroids or hyaluronic acid injection within 3 months preceding enrollment;
8. Serious uncontrolled medical conditions such as cancer, uncontrolled cardiovascular disorder, severe hepatic/renal insufficiency, or coagulation disorder;
9. Known allergy, contraindication, or intolerance to diclofenac, acetaminophen, or gel components;
10. Known phobia to acupuncture or having received acupuncture treatment within 4 weeks prior to enrollment.

### Interventions

#### EA group

The EA protocol was determined according to the meridian theory of TCM and the consensus of three senior doctors of TCM who have more than 30 years’ clinical experience. The acupuncturists who will perform the interventions will be state-licensed and have more than 2 years’ practical experience.

We will apply Ashi points (the most obvious tender points over the affected joints), Baxie points (Ex-UE9, 4 points proximal to the margin of the webs between each two of the five fingers of a hand), Houxi (SI3), and Waiguan (TE5) in this trial. The location of the above acupoints will be based on the nomenclature and location of acupuncture points drafted by the National Standard of the People’s Republic of China (GB/T 12 346-2006) [25]. Sterile single-use stainless steel needles (size 0.3 mm × 40 mm) and the SDZ-V EA apparatus (all Hwato brand, Suzhou Medical Appliance Factory, Suzhou, China) will be utilized. After local skin disinfection with 75% alcohol wipes while patients are in a sitting or supine position, acupuncturists will insert needles perpendicularly into the Ashi points to a depth of approximately 2–3 mm until they pierce the bone surface; horizontally into the Baxie points toward the wrist to a depth of 5–10 mm; and perpendicularly into SI3 and TE5 to a depth of approximately 5–10 mm. The depth of needling will vary based on the participant’s body sizes. After insertion, all needles except those in the Ashi points will be manually manipulated to achieve De qi sensation, which is defined as a composite of unique needling sensations, including soreness, numbness, distention, or heaviness felt by both participants and acupuncturists [26]. Thereafter, the needles at Baxie 1 (between thumb and index fingers) and Baxie 4 (between the ring finger and little finger) will be connected using alligator clips from the EA apparatus. The electrical stimulation will last for 30 min with a continuous wave of 10 Hz and a fixed current intensity of 0.5–2 mA according to the participants’ sensitivity. All the needles will be retained for 30 min and then gently removed.

Participants will undergo EA treatment three times per week for a total of 12 sessions in 4 consecutive weeks.

#### Topical DSG group

Participants will be shown how to apply the topical DSG (Mayinglong Pharmaceutical Group Co., Ltd., Hubei, China; 100 mg of diclofenac sodium per gram of gel, 20 g in each tube; H19990010) under supervision and will be provided with detailed instructions for the use and application of the gel as well as dosing pads to standardize the application amount. The gel will be gently rubbed over the affected joints without excessive joint movement four times per day for 4 weeks, and participants will be asked not to wash their hands for 1 h after application. Each hand treatment will be used in identical dispensing doses of gel (4-cm strip, approximately 2 g, which was judged sufficient for approximately half the surface of each hand [200 cm^2^] [15]). Dosing times should be distributed evenly over waking hours.

If bilateral hands are affected, both sides will be treated. The value in the dominant hand or both hands will be used in the analysis according to the requirements of different outcome measurements.

### Rescue medication

Only acetaminophen (500 mg/T, Tianjin Smith Kline & French Laboratories Ltd., Tianjin, China) will be allowed to a maximum dose of 2 g daily for up to 3 days as rescue medication if insufferable pain occurs. Participants will not be allowed to take rescue medication within 60 h before the baseline and outcome measurements. If a participant takes rescue medication within the prohibited time during the treatment and follow-up period, the assessor will postpone the outcome measurement visit.

### Outcome measures

#### Primary outcome

The primary outcome in this trial is the change in average overall finger joint pain intensity in the dominant hand over the previous 48 h, measured on a 0–100 VAS (0 = no pain, 100 = maximal pain) from baseline to week 4. The changes at week 8 and week 16 from baseline also will be assessed.

#### Secondary outcomes

The secondary outcomes are as follows:

1. The proportion of participants achieving at least a 15-point reduction in average overall finger joint pain intensity in the dominant hand from baseline to weeks 4, 8, and 16: As previously described, 15 of 100 mm for absolute improvement could reflect a minimum clinically important improvement in pain for patients experiencing hand OA [27].
2. Change in Australian Canadian Osteoarthritis Hand Index (AUSCAN) total score, and pain, stiffness, and physical function subscales from baseline to weeks 4, 8, and 16: The AUSCAN index contains a 15-item scale referring to hand pain (5 items), stiffness (1 item), and function (9 items) during the preceding 48 h [28], which is valid, reliable, and responsive in patients with hand OA [29]. All items are scaled on a 0–100 VAS (0 = none to 100 = very severe), with higher scores indicating more severe symptoms/functional impairment. The AUSCAN functional assessments will be performed for the dominant hand only. The total AUSCAN score will be calculated as the average of scores on 15 questions [29].
3. Change in Functional Index for HOA (FIHOA) [30] from baseline to weeks 4, 8, and 16: The FIHOA is a patient-reported hand function questionnaire, comprising 10 items with a total score is calculated, ranging from 0–30 (0 = best activity performance, 30 = very poor activity performance).
4. Change in the number of self-reported painful joints and painful joints at digital pressure and swollen joints from baseline to weeks 4, 8, and 16.
5. Change in hand grip strength and pinch strength of the fingers from baseline to weeks 4, 8, and 16: Hand grip strength and pinch strength of the fingers of the dominant hand will be tested using a hand dynamometer (KYTO 2324, Kangdu Electronic Manufacture Co. Ltd, Dongguan, China) and a Jamar digital pinch gauge (Allegro Medical, Illinois, USA), respectively. Grip strength will be measured first, followed by pinch strength of the fingers, including palmar (three-jaw chuck) pinch, key (lateral) pinch, and tip (two-point) pinch [31]. For each of the tests, the participants will be in a standing position with the shoulder adducted and neutrally rotated and the elbow slightly extended [32]. Participants will conduct three trials, with an interval of 30 s at a minimum between each trial. The final score for analysis will be recorded in kilograms, and the average of the three consecutive values measured.
6. Change in patient global assessment of improvement [33] from baseline to weeks 4, 8, and 16: The patients will be asked to respond to the question “Considering all the ways your hand OA affects you, how have you been during the last 48 h?” [34] on a self-administered 0–100 VAS (0, worst possible, to 100, best possible, in 10-point increments) at 4, 8, and 16 weeks.
7. The proportion of responders according to the Outcome Measures in Rheumatological Clinical Trials (OMERACT) and Osteoarthritis Research Society International (OARSI) responder criteria [35] at weeks 4, 8, and 16: The OMERACT-OARSI response is defined as an improvement in either pain (0–100 overall finger joint pain) or function (0–100 AUSCAN physical function) by ≥ 50% (relative) and ≥ 20/100 (absolute); or an improvement of ≥ 20% (relative) and ≥ 10/100 (absolute) in ≥ 2 of the following: pain, functioning, and patient global assessment of improvement.
8. Change in quality of life assessed by the World Health Organization Quality of Life abbreviated version (WHOQOL-BREF) from baseline to weeks 4, 8, and 16: The WHOQOL-BREF is a 26-item self-report questionnaire rated on a 5-point Likert-type scale with four domains of QOL: physical (7 items), psychological (6 items), social (3 items), and environment (8 items), plus two items representing the general QOL [36]. The four domain scores are scaled in a positive direction with higher scores indicating a higher QOL. In this trial, we will use the Chinese version of the WHOQOL-BREF, which has been reported to have adequate reliability and validity [37].
9. Participants’ expectations for acupuncture at baseline: At baseline, participants in the EA group will be asked to answer the following question: “What level of improvement do you expect from EA for your hand OA?” Participants can choose one of the following five answers: “extreme improvement,” “moderate improvement,” “slight improvement,” “no improvement,” or “unclear.”
10. Self-reported consumption of acetaminophen for hand OA during weeks 1–4 and weeks 5–12: the proportion of participants using acetaminophen and the average dosage of acetaminophen used weekly will be calculated and assessed.

#### Safety assessment

Any AEs occurring throughout the complete study period, whether reported spontaneously by the participants or observed by the researchers, will be recorded and categorized as acupuncture-related AEs (e.g., fainting, broken needle, localized hematoma, dizziness), a side effect of TDG (e.g., skin irritation/itching, reddening, scaly skin), or a non-treatment–related AE. All AEs will be recorded in detail using a case report form.

#### Sample size calculation

This trial hypothesizes that 4 weeks of EA treatment will be superior to topical DSG in terms of pain relief in the dominant hand for patients with hand OA. Based on the results of a previous study [15], the OA pain intensity in the dominant hand on the 0–100 VAS scale was reduced by an average of 31.1 points in the topical DSG group and 23.9 points in the vehicle group. We predict that EA will display superiority over topical DSG by at least a 7-point better relative reduction in VAS score with a standard deviation of 12. Assuming a two-tailed test with a possible drop-out of 10% of the patients, a total of 108 patients (54 in the EA group and 54 in the topical DSG group) will be required for 80% power on a 5% alpha level.

#### Statistical analysis

The null hypothesis is that the change from baseline in the average overall finger joint pain intensity in the dominant hand at week 4 will be the same for the EA group and the topical DSG group. Data for continuous variables will be presented as means with standard deviations or medians with interquartile ranges, and for categorical variables, the data will be shown as frequencies (number of cases) and relative frequencies (percentages). For analysis of normally distributed quantitative variables, repeated-measures analysis of variance (ANOVA) will be used, while for the data of the groups with abnormal distribution, a nonparametrical Kruskal–Wallis test will be performed. Categorical variables will be analyzed using the Chi-square (*χ*^2^) test. Two-tailed *P* value < 0.05 is considered statistically significant. All outcome variables will be analyzed on an intention-to-treat (ITT) principle with an all randomized population included using SPSS software V.26.0 (IBM SPSS Statistics; IBM Corp, Somers, NY). We will impute missing primary outcome data by using multiple imputation techniques under the missing at random assumption for the ITT population, and we will not impute missing secondary outcome data.

### Quality control

All participating staff will attend uniform training before the commencement of the trial to ensure the quality of the trial. The licensed acupuncturists who have a minimum of 2 years’ clinical practice of acupuncture will perform the treatment. Throughout the trial, the principal investigator and a research coordinator will regularly check CRFs, acupuncture operation, patient safety, and data quality. Withdrawals or study dropouts will be clearly described during the trial.

To minimize compliance bias in the topical diclofenac sodium gel group, we will dispense the gel with uniform specifications and provide dosing pads to standardize the applying amount of the diclofenac gel. Diary cards will be also supplied including the exact date and time when using the gel and the dose per use, which are required to be recorded every day by patients. The outcome evaluator will check the returned tubes once the new gel is supplied.

## Discussion

Hand OA is an increasingly prevalent condition among older adults, and it is associated with a serious disease burden [38]. Currently available interventions for hand OA have several limitations, and other analgesic approaches are still needed. Acupuncture has been considered a promising therapy in clinical practice for the management of various pain-related conditions including knee OA [17] with a good safety profile. However, there is a distinct lack of studies assessing the effect of acupuncture for hand OA. Considering the ethics and acceptance by participants, we chose topical DSG, which has been recommended as the preferred treatment option, as an active comparator in this trial. The results of this trial will help determine which of the two treatment protocols, EA or topical DSG, is more effective for the treatment of hand OA in the clinic.

Because measurement of pain is a core outcome domain in hand OA clinical trials [34], and the VAS has been agreed upon as the recommended preliminary instrument for assessing pain [39] with good validity and responsiveness [40], we chose the change in average overall finger joint pain intensity in the dominant hand over the preceding 48 h, measured on a 0–100 mm VAS from baseline to week 4 as the primary clinical endpoint in this study.

Moreover, the outcomes for other core domains (i.e., physical function, joint activity, hand strength, patient global assessment, and health-related QOL) endorsed by OMERACT12 [34] will also be assessed in this trial. The results will provide detailed information regarding the effectiveness of EA as a treatment for hand OA.

The strengths of the trial include the strict inclusion and exclusion criteria, measurement of outcomes recommended by the OMERACT-OARSI, and blinding of the outcome assessor and data manager. We acknowledge several limitations exist in this trial. First, although this study could provide clinically relevant data important for patients with hand OA and the clinicians who treat them, the absence of a comparator group treated with placebo will make it difficult to exclude non-specific physiological responses to acupuncture. Second, we will be not able to blind participants and acupuncturists, which could potentially introduce bias in the results. Third, although symptomatic improvement may occur secondary to structural benefit, we will not use hand imaging assessments, because we do not expect our treatment to bring a structural change in the short term. Fourth, topical DSG will be used four times daily in our study based on evidence from previous literature [15, 41] and clinical experience. However, it may lead to a relatively poor compliance for patients, and result in a certain compliance bias. Fifth, considering the forces acting on the first CMC joint may much higher than that on the DIP and PIP joints in daily life, the comparison of the numbers of joints involved between patients would bring bias; the result will be interpreted as exploratory and viewed with caution. Sixth, the fixed acupuncture regimen will be used in this study; however, whether different acupuncture doses or different acupoints will show different benefits for patients should be evaluated in future studies.

## Data Availability

Data are available upon reasonable request.
